# Altered Lactylation Myocardial Tissue May Contribute to a More Severe Energy-Deprived State of the Tissue and Left Ventricular Outflow Tract Obstruction in HOCM

**DOI:** 10.3390/bioengineering12040379

**Published:** 2025-04-03

**Authors:** Ruoxuan Li, Jing Wang, Jia Zhao, Jiao Liu, Yuze Qin, Yue Wang, Yiming Yuan, Nan Kang, Lu Yao, Fan Yang, Ke Feng, Lanlan Zhang, Shengjun Ta, Bo Wang, Liwen Liu

**Affiliations:** Department of Ultrasound Medicine, Xijing Hospital, The Fourth Military Medical University, Xi’an 710032, China; lrx.friend5@163.com (R.L.); wangjing2015@fmmu.edu.cn (J.W.); 18292876991@163.com (J.Z.); summer410119@163.com (J.L.); dzdy123456@163.com (Y.Q.); wangyuejinnn@163.com (Y.W.); 17535901718@163.com (Y.Y.); kangnan369@163.com (N.K.); 15101165016@163.com (L.Y.); amig_092587@163.com (F.Y.); kfengrex@163.com (K.F.); lanlzh1207@163.com (L.Z.); shengjunt@126.com (S.T.)

**Keywords:** hypertrophic cardiomyopathy, obstructive, left ventricular outflow tract obstruction, lactylation, energy metabolism

## Abstract

Hypertrophic cardiomyopathy (HCM) is the most common hereditary cardiovascular disease. In general, obstructive hypertrophic cardiomyopathy (HOCM) is more closely related to severe clinical symptoms and adverse clinical outcomes. Therefore, it is necessary to explore the possible causes of HOCM, which may help physicians better understand the disease and effectively control and manage the progression of the disease. In recent years, the discovery of lactylation has provided scholars with a new direction to explore the occurrence of diseases. In cardiovascular diseases, this post-translational modification can exacerbate cardiac dysfunction, and it can also promote the cardiac repair process after myocardial infarction. In this study, we used the myocardial tissue of mice carrying the *Myh7* V878A gene mutation site for protein lactylation detection. Through a further analysis of the enriched pathways using KEGG enrichment, GO enrichment, and Wiki Pathways enrichment, we found that the enriched pathways with lactylation modifications in the HOCM mice mainly included the fatty acid oxidation pathway, the tricarboxylic acid cycle pathway, the adrenergic signaling pathway in cardiomyocytes, and the cardiomyocyte hypertrophy pathway. Among the above pathways, significant changes in lactylation occurred in proteins including Acads, Acaa2, Mdh2, Myl2, and Myl3. We used the COIP experiment to verify the omics results and the ELISA assay to verify the function of the enzymes. We found that a decrease in lactylation modifications also led to a decrease in enzyme function. The abnormalities of these proteins not only lead to abnormalities in energy metabolism in the myocardial tissue of HOCM but also may affect myocardial contractility, resulting in the impaired contractile function of HOCM. The results of this study lay a preliminary theoretical foundation for further exploring the pathogenesis of HOCM.

## 1. Introduction

Hypertrophic cardiomyopathy (HCM) is the most common inherited cardiovascular disease with a prevalence about 1 in 500 to 200, and is the leading cause of sudden cardiac death in adolescents and athletes [1,2,3]. In general, obstructive hypertrophic cardiomyopathy (HOCM), as one of the most important subtypes of HCM, is more closely related to severe clinical symptoms and adverse clinical outcomes such as atrial fibrillation, heart failure, and malignant ventricular arrhythmias. Therefore, it is necessary to explore the possible causes of HOCM, which may help physicians better understand the disease and effectively control and manage the progression of the disease.

At present, most scholars explore the occurrence of left ventricular outflow tract obstruction (LVOTO) from the perspective of structural changes, but the causes of structural abnormalities have not been fully elucidated [4,5]. Previous studies have shown that HOCM patients are more dependent on glycolysis for energy production, resulting in a greater accumulation of lactate in the body [6,7,8]. The role of lactate has been well studied in other cardiovascular diseases. For instance, studies have found that the abnormal lactylation of the α-myosin heavy chain is related to the occurrence of heart failure, and the pyruvate–lactate axis can regulate the occurrence of myocardial hypertrophy and heart failure [9,10]. Moreover, the alteration of metabolism precedes the appearance of a clinical phenotype [11,12], especially for heart failure, which causes similar metabolic changes to those seen in HCM patients. We speculate that lactate also plays a role in the pathogenesis and energy metabolism of HOCM. Due to the difficulty of obtaining human heart samples, we selected C57 mice with the *Myh7* V878A gene mutation, which is a homologous gene to that in humans, used for testing [13].

The purpose of this study was to detect the differences in lactylation in the myocardial tissues of HOCM and non-obstructive hypertrophic cardiomyopathy (NOHCM) C57 mice carrying *Myh7* V878A gene mutations to preliminarily explore the metabolic factors related to the occurrence of HOCM and to search for key proteins altered in key pathways in order to help doctors and scholars to further understand HOCM and to search for therapeutic targets.

## 2. Material and Methods

### 2.1. Mice Screening and Sample Preparation

#### 2.1.1. Genetic Testing of Mice

Six-month-old C57 mice carrying the *Myh7* V878A gene mutation and wild-type (WT) C57 mice were used. The genotypes were identified by sequencing using the following primer pairs: 3′-GCCGCAAGGAGCTGGAGGAGAAGATGGcGTCtCTcCTGCAGGAGAAGAATGACCTGCAGCTCCAAG-5′.

#### 2.1.2. Basic Information Acquisition and Echocardiography of Mice

After measuring the body weight of each mouse, the mice were subjected to echocardiography. Echocardiography was performed on mice anaesthetized with isoflurane to measure left ventricular ejection fraction (LVEF), maximum wall thickness of the left ventricle (MLVWT), interventricular septum diameter in end-diastole (IVSd), and left ventricular outflow tract pressure gradient (LVOT-PG) using the VINNO 9 laboratory model. M-mode ultrasound images were measured in the short axis plane of the high left ventricular papillary muscle on which LVEF was measured. LVOT-PG was measured in left ventricular long axis section. In the present study, C57 mice carrying the *Myh7* V878A gene mutation with LVOT-PG > 11.1 mmHg and the presence of MLVWT exceeding normal values (IVSd 0.71 ± 0.15 mm, LVPWd 0.79 ± 0.22 mm) were considered as HOCM mice. C57 mice carrying the *Myh7* V878A gene mutation with MLVWT exceeding normal values but LVOT-PG ≤ 11.1 mmHg were considered to be NHOCM mice. Mice that had not been artificially domesticated and whose heart measurements were within the normal range were WT mice. The above thresholds and measurements were based on previous studies and guidelines [14,15]. After recording echocardiographic information, mice were sacrificed and tibias were stripped and measured.

#### 2.1.3. Myocardial Tissue Sample Preparation of Mice

We collected myocardial tissues from HOCM and NOHCM C57 mice carrying the *Myh7* V878A gene mutation and performed proteomic analysis to assess protein lactylation. Hearts were removed and immediately placed in pre-cooled PBS to wash the blood and remove non-cardiac tissue. PBS was blotted from the surface of the heart with absorbent paper and placed in a sample tube in liquid nitrogen for 5 min and stored at −80 °C. The samples were thawed at 4 °C and 80 mg of each sample was mixed with 25 μL isotope internal standards and 400 μL of cold methanol/acetonitrile solution (1:1, *v*/*v*). The lysate was homogenized by MP homogenizer (20 s, thrice), adequately vortexed, then ultrasounded for 5 min at low temperature, followed by incubation at −20 °C for 1 h. The mixture was centrifuged for 20 min (14,000 rcf, 4 °C). The supernatant was taken over the Ostro plate, and the receiving solution was divided into two tubes (one tube is 2/3 and the other tube is 1/3). Two-thirds of the receiving solution was dried in a vacuum centrifuge, the samples were re-dissolved in 150 μL acetonitrile/water (1:1, *v*/*v*) and adequately vortexed, and then centrifuged (14,000 rcf, 4 °C, 15 min). The supernatants were collected for LC-MS/MS analysis.

**Figure 1:**
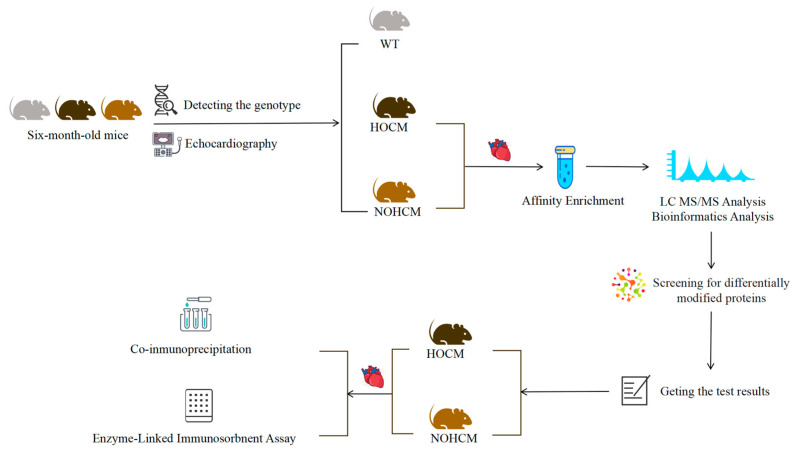
Study design workflow.

This study was approved by Welfare and Ethics Committee of Laboratory Animal Centre of the Fourth Military Medical University (No. IACUC-2020151). All experimental procedures and animal welfare protocols adhered to the National Institute of Heath guidelines for the care of laboratory animals. The workflow of this study is shown in Figure 1.

Detailed experimental procedures including protein extraction, trypsin digestion, affinity enrichment, and liquid chromatography-tandem mass spectrometry analysis can be found in Supplementary Material.

### 2.2. Co-Immunoprecipitation (Co-IP) and Western Blot Analysis

After extracting the proteins and reserving the Input group, the immune complexes were extracted using Thermo’s Pierce™ Classic Magnetic IP/Co-IP Kit (No. 88804) (Thermo Fisher Scientific, Waltham, MA, USA) according to the instructions. A mass of 40 μg of total protein was boiled in 5× SDS buffer for 10 min, subjected to SDS-PAGE electrophoresis, and electrotransferred to a nitrocellulose membrane. The nitrocellulose membrane was covered with TBST containing 50 g/L skimmed milk powder at room temperature for 1.5 h; then, the corresponding primary antibody at 1:1000 was added dropwise sequentially, and the membrane was washed with TBST 3 times, each time for 10 min, at 4 °C, and left overnight. Then, the membrane was incubated with the secondary antibody at 1:5000 for 2 h, and then washed with TBST 3 times, each time for 10 min. Finally, the membrane was exposed to ECL luminescent solution for developing, and the Bio-RAD photographic system was used for photographic and electrophotographic development and electrophotographic transfer of 40 μg of total proteins. The RAD camera system was used to photograph and analyze the relative expression of the proteins using Image Lab software (version: 6.1/6.0). The detailed CO-IP experimental procedure can be found in the Supplementary Materials.

### 2.3. Enzyme-Linked Immunosorbent Assay (ELISA)

The antibody was diluted with carbonate coating buffer to a protein content of 1–10 μg/mL. A volume of 100 μL per well was added to a polystyrene plate at 4 °C and left overnight. The next day, the solution in the wells was discarded and these were washed 3 times with wash buffer for 3 min each time. A volume of 200 μL of blocking solution was added to each well and this was incubated at 37 °C for 1–2 h. The sealing film was carefully removed, put in the microplate washer, and washed 3–5 times. A volume of 100 μL of the appropriately diluted sample to be tested was added to the coated reaction wells described above. The plate was sealed with a film and incubated at 37 °C for 1–2 h. This was washed again. A volume of 100 μL of diluted biotinylated antibody working solution was added to each well, and incubated at 37 °C for 1 h after sealing with sealing film. This was washed again. A volume of 100 μL of diluted biotinylated antibody working solution was added to each well, and incubated at 37 °C for 30 min after sealing with sealing film. A volume of 100 μL of TMB substrate solution was added to each well, and the reaction was carried out at 37 °C in the dark for 10~30 min until the wells of the diluted standard showed a clear color gradient. A volume of 100 μL of 2 M sulfuric acid was added to each reaction well, and the color changed from blue to yellow. Within 10 min, the OD value of each well was measured at 450 nm on the microplate reader. The sample concentration was calculated from the sample curve.

### 2.4. Statistical Analysis

#### 2.4.1. Omics Data Analysis

The R language was used for omics data analysis. Two statistical analysis methods were used to evaluate repeatability including Pearson’s correlation coefficient (PCC) and pareto-scaled principal component analysis (PCA). Univariate statistical analysis methods used in this study included Fold Change Analysis (FC Analysis) and T-test/Fisher’s exact test to analyze the significance of functional enrichment of differentially expressed proteins (using the identified protein as the background). Functional terms with *FC* > 1.5 or *FC* < 0.67 and *p* value < 0.05 were considered significant.

Enrichment pathway analysis was performed using Kyoto Encyclopedia of Genes and Genomes (KEGG) enrichment (https://www.kegg.jp/) (accessed on 20 May 2024), GO enrichment (https://www.geneontology.org/) (accessed on 20 May 2024), and Wiki Pathways enrichment (https://www.wikipathways.org/) (accessed on 22 May 2024).

#### 2.4.2. Basic and Experimental Data Analysis

SPSS 27.0 software was used for statistical analysis of basic and experimental data of mice. Continuous variables conforming to normal distribution were represented by $\bar{X}\pm S$ and analyzed using Student’s *t*-test and one-way ANOVA. Continuous variables that did not fit normal distribution were represented by median (Q1, Q3) and analyzed using Mann–Whitney U-test. In this study, *p* < 0.05 was statistically significant.

## 3. Results

### 3.1. Basic Information and Echocardiography of Mice

**Figure 2:**
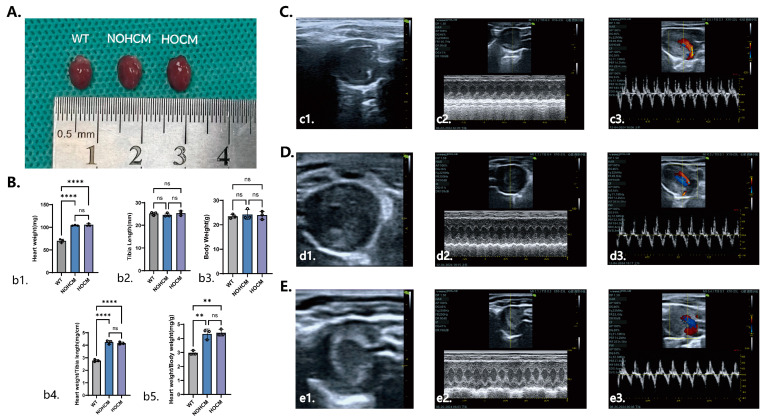
Cardiac samples and ultrasound images of HOCM mice, NOHCM mice, and WT mice. (**A**) Cardiac samples of HOCM mice, NOHCM mice, and WT mice. (**B**) Cardiac sample charts: (**b1**) heart weight; (**b2**) tibia length; (**b3**) body weight; (**b4**) heart weight/tibia length; (**b5**) heart weight/body weight. (**C**) Echocardiography of WT mice: (**c1**) echocardiography of short axis section of the left ventricle; (**c2**) echocardiography of M-mode; (**c3**) echocardiography of left ventricular long axis section to measure LVOT-PG. (**D**) Echocardiography of NOHCM mice: (**d1**) echocardiography of short axis section of the left ventricle; (**d2**) echocardiography of M-mode; (**d3**) echocardiography of left ventricular long axis section to measure LVOT-PG. (**E**) Echocardiography of HOCM mice: (**e1**) echocardiography of short axis section of the left ventricle; (**e2**) echocardiography of M-mode; (**e3**) echocardiography of left ventricular long axis section to measure LVOT-PG. HOCM, hypertrophic obstructive cardiomyopathy; NOHCM, non-obstructive hypertrophic cardiomyopathy; WT, wild-type; LVOT-PG, left ventricular outflow tract pressure gradient. ns, *p* > 0.05; ** *p* < 0.01; **** *p* < 0.0001.

The HOCM mice, NOHCM mice, and WT mice were all male and placed in the same cage at 6 months of age; all of them had the same body weight (*p* = 0.844) and tibia length (*p* = 0.576). Compared to the WT mice, the HOCM mice and NOHCM mice had a higher heart weight (*p* < 0.05), ratio of heart weight and body weight (*p* < 0.05), ratio of heart weight and tibia length (*p* < 0.05), and MLVWT (*p* < 0.05). Compared to the WT mice and HOCM mice, the NOHCM mice had a higher LVEF (*p* < 0.05). Compared to the WT mice and NOHCM mice, the HOCM mice had a higher LVOT-PG (*p* < 0.05) (Table 1; Figure 2).

### 3.2. Lactylation of Mouse Myocardial Tissue

**Figure 3:**
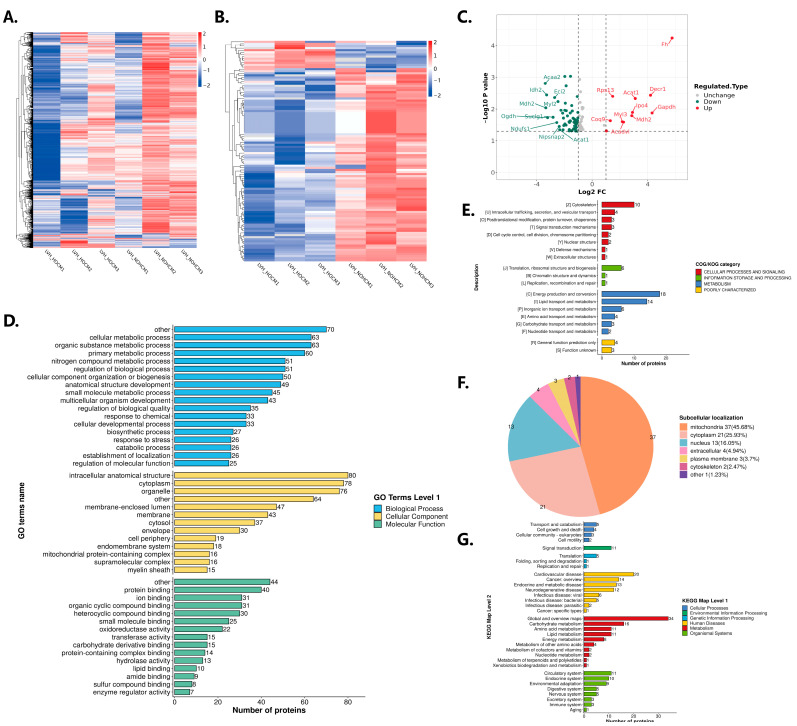
Comprehensive evaluation of lactylation results. (**A**) Heatmap of all lactylation proteins in HOCM and NOHCM groups. (**B**) Heatmap of all differential lactylation proteins in HOCM and NOHCM groups. (**C**) Volcano plots of statistically significant levels of lactylation for these proteins among HOCM and NOHCM groups. (**D**) GO secondary classification for analyzing the function of differentially modified proteins. (**E**) COG/KOG functional classification for analyzing the function of differentially modified proteins. (**F**) Subcellular localization classification for analyzing the function of differentially modified proteins. (**G**) KEGG functional classification for analyzing the function of differentially modified proteins.

A total of 2710 proteins and 105 lactylation sites were identified in this study (Figure 3A). The PCC and PCA showed a better correlation and reproducibility of the samples (Figure S1A,B). The overlap in the PCA may be due to the fact that the two groups were mice of the same age carrying the same type of mutation and both had the HCM phenotype. The LVOT-PG and LVEF were significantly different between these two groups. In the HOCM mice’s myocardial tissues, there were 12 lactylation up-regulation sites with 12 up-regulated lactylated proteins and 93 lactylation down-regulation sites with 73 down-regulated lactylated proteins (Figure 3B). The volcano plots showed statistically significant levels of lactylation for these lactylated proteins among the HOCM and NOHCM groups (Figure 3C). The analysis of the function of differentially modified proteins using GO secondary classification, subcellular localization classification, COG/KOG functional classification, and KEGG functional classification revealed that the differentially modified proteins were mainly focused on energy metabolism processes in mitochondria (Figure 3D–G).

**Figure 4:**
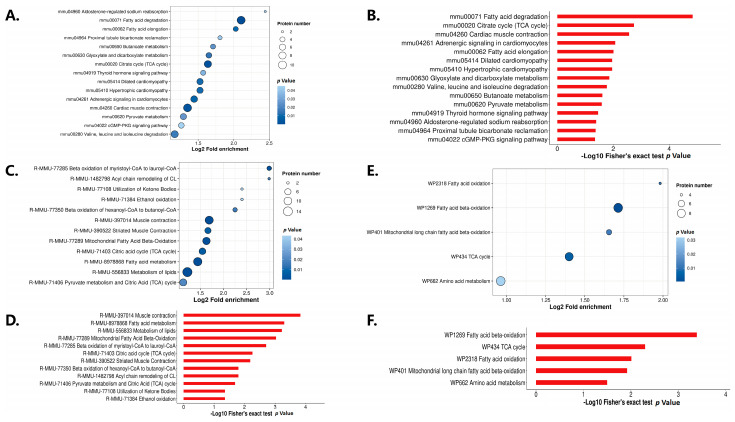
Analysis of enriched pathways of lactylation. (**A**,**B**) KEGG pathway for enrichment analysis. (**C**,**D**) Reactome for enrichment analysis. (**E**,**F**) WikiPathways for enrichment analysis.

After that, we performed an enrichment analysis at three levels including the KEGG pathway (Figure 4A,B), Reactome (Figure 4C,D), and WikiPathways (Figure 4E,F). We found that the differentially expressed modified proteins were mainly concentrated in fatty acid metabolism and the tricarboxylic acid cycle (TCA). Meanwhile, in the analysis of the KEGG enrichment pathway, we found that, in addition to significant changes in energy metabolism-related pathways, the hypertrophic cardiomyopathy pathway and adrenergic signaling in the cardiomyocyte pathway were also significantly changed.

### 3.3. Experimental Verification of Lactylation of Proteins

**Figure 5:**
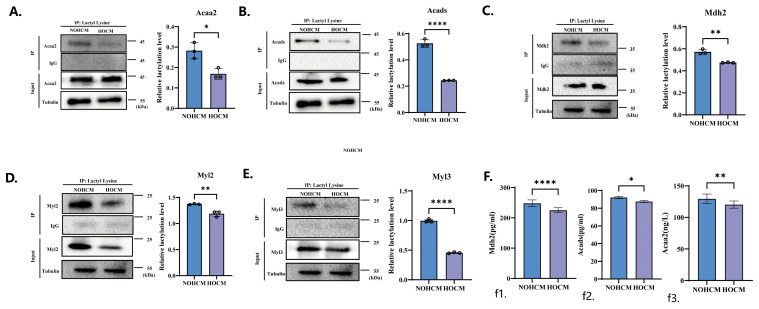
Experimental verification results of enzymes and proteins that are significantly modified by lactylation. (**A**–**E**) Hearts from HOCM mice and NOHCM mice were lysed and immunoprecipitated with Acaa2 antibody (**A**), Acads antibody (**B**), Mdh2 antibody (**C**), Myl2 antibody (**D**), Myl3 antibody (**E**) or control IgG, followed by detection of lactyl lysine. (**F**) Functional verification of enzymes Mdh2 (**f1**), Acacds (**f2**), and Acaa2 (**f3**). * *p* < 0.05; ** *p* < 0.01; **** *p* < 0.0001.

We screened the most significantly lactated proteins in the myocardial tissues of HOCM mice compared to NOHCM mice. We found that in the analysis of the KEGG pathway, Reactome and WikiPathways, two proteins including malate dehydrogenase 2 (Mdh2) (Figure 5C) and acyl-CoA dehydrogenase short chain (Acads) (Figure 5B) were lactated in the fatty acid metabolism pathway and TCA pathway. Importantly, acetyl-CoA acyltransferase 2 (Acaa2) (Figure 5A) and Mdh2 were the most significant proteins with lactylation and the HOCM group was down-regulated by 10-fold compared with the NOHCM group. Although there is no Acaa2 in the analysis of WikiPathways and the down-regulation ratio is only about 2-fold, this protein has a clear position in the analysis of the KEGG pathway and Reactome. Meanwhile, in the hypertrophic cardiomyopathy pathway and adrenergic signaling in cardiomyocyte pathway, which are closely related to the occurrence of hypertrophic cardiomyopathy, myosin Light Chain 2 (Myl2) and myosin Light Chain 3 (Myl3) were the most significant proteins with lactylation (Figure 5D,E).

Due to the important roles of Acads and Acaa2 in fatty acid metabolism, as well as the significant functions of Mdh2 in the TCA cycle and the malate–aspartate shuttle, we verified the functions of these three enzymes. We found that compared with those in the NOHCM mice, the functions of Acads, Acaa2, and Mdh2 in the myocardial tissues of the HOCM mice were significantly reduced (Figure 5F).

## 4. Discussion

At present, more and more studies have found that there are significant metabolic disorders in HCM, especially glucose metabolism disorders [6,8,16]. Lactate, as the main product of glycolysis, used to be considered as a metabolic waste product that did not have any major physiological functions [17,18]. But with the deepening of research, the function of lactate has gradually been revealed and proven [9,19]. In this study, we selected HCM mice carrying the *Myh7* V878A gene mutation to preliminarily explore the reasons for energy deficiency in HOCM mice and the possible causes of the disease. We found that some proteins in the HOCM mice underwent significant lactylation and that these proteins were concentrated in metabolic pathways and key pathways leading to the development of HCM, which may contribute to myocardial metabolic disorders and the development of HCM.

### 4.1. The Mouse Model Used in This Study Carried the Same Mutation Site as HCM Patients Which Can Simulate HCM Patients Well

In this study, we used C57 mice carrying the *Myh7* V878A gene mutation, which is a hot spot among ethnic Han Chinese individuals and has a high penetrance [20]. A functional analysis of the conserved sequences suggested that this mutation may cause significant alteration of its function [13,20]. We performed echocardiography on the mice carrying this mutation and measured the basal data.

We found that the C57 mice carrying the *Myh7* V878A gene mutation had an HCM phenotype similar to that of humans. Compared with the WT mice, the C57 mice carrying this gene mutation exhibited cardiac enlargement, an increase in the MLVWT, enhanced cardiac systolic function, and an elevation in the LVOT-PG. These findings indicate that C57 mice carrying the *Myh7* V878A gene mutation are capable of mimicking the phenotype of human hypertrophic cardiomyopathy and can be used for subsequent research.

### 4.2. Differences in Lactylation of Myocardial Tissue Between HOCM and NOHCM Mice May Relate to Abnormal Energy Metabolism and the Development of LVOTO

It has been confirmed that HOCM patients have more lactate in their bodies compared to NOHCM patients in previous study [8]. However, there is still a lack of exploration of the role of lactate in HOCM. In our study, we found significant alterations of the lactylation of proteins including Acads, Acaa2, Mdh2, Myl2, and Myl3. Among these proteins, Acads, Acaa2, and Mdh2 are key enzymes in energy metabolism, whereas Myl2 and Myl3 are closely related to the development of HCM [21,22,23]. In detail, first of all, Acads and Acaa2 play important roles in fatty acid β-oxidation, in which Acads can catalyze the first step of mitochondrial fatty acid fatty acid β-oxidation and Acaa2 is one of the enzymes that catalyze the last step of this process. Our study has confirmed that the functions of Acads and Acaa2 in HOCM mice are reduced compared to those in NOHCM mice. In previous studies, the overexpression of ACAA2 inhibited triglyceride production in mammary epithelial cells [24]. The reduced expression of ACAA2 impaired fatty acid β-oxidation and eventually exacerbated kidney fibrosis in acute kidney injury [25]. Biallelic variation in the ACADS gene can cause short-chain acyl CoA dehydrogenase deficiency, which can lead to a variety of phenotypes including fatty acid oxidation disorders [26]. Therefore, abnormalities of Acads and Acaa2 may be the main reason for fatty acid metabolism disorder in the myocardial tissue of HOCM mice and may be associated with the occurrence of fibrosis, which is one of the most significant changes in HCM patients, especially in patients with HOCM [6,8,16]. Secondly, Mdh2 is localized to the mitochondria and may play pivotal roles in the malate–aspartate shuttle process, which is closely related to the occurrence of myocardial hypertrophy [27]. A previous study has confirmed that mitochondrial TCA cycle enzymes play an important role in epigenetic regulation [28]. Our study has confirmed that the function of Mdh2 is reduced in HOCM mice compared to that in NOHCM mice. This can lead to a decrease in the malate–aspartate shuttle, prompting pyruvate to accept hydrogen ions and convert into lactate, resulting in lactate accumulation in the myocardium. This could further impair the energy metabolism process of myocardial cells and cause abnormalities of energy metabolism. According to previous studies, abnormal myocardial energy metabolism is not only related to the occurrence of myocardial hypertrophy, but, also, the myocardial hypoxia state caused by it can damage the myocardium and induce heart failure [6,29]. Since normal myocardium mainly relies on fatty acids for energy production, impaired fatty acid metabolism can cause changes in the heart’s energy production pattern [6]. Some metabolites are closely related to the occurrence of adverse clinical events in patients, resulting in abnormally high cardiac damage indicators in patients, which are closely related to the assessment of patients’ conditions and prognosis [11,12,30,31,32]. Thirdly, although the association of Myl2 and Myl3 with the occurrence of HCM has been demonstrated, the cause of Myl2 and Myl3 abnormalities is unknown and their association with HOCM has not been well studied [21,22,33]. In our study, it was found that the lactylation of Myl2 and Myl3 were altered in the myocardial tissues of the HOCM and NOHCM mice and the degree of lactylation was different between the two groups, which may be the cause of the dysfunction of Myl2 and Myl3 and, in turn, leads to the occurrence of the disease. Abnormalities in the two proteins can affect the function of cardiac myosin, especially the myosin light chain, which plays an important role in ATPase activity and affects energy production. Importantly, abnormalities of cardiac myosin can affect myocardial contractility, which may be the result of the more severe impaired myocardial contractility in HOCM. Generally, the myocardial contractility in HCM patients is increased [3]. In this study, the echocardiography of the HOCM mice also clarified this clinical manifestation. The myocardial contractility of the NOHCM mice was enhanced compared to that in WT mice and HOCM mice, while there was no significant difference in the myocardial contractility between the HOCM mice and WT mice. In the HOCM mice, their systolic function was relatively reduced.

In conclusion, our study has shown that significant lactylation occurs in the enzymes and proteins within the myocardium of HOCM mice. This process may lead to abnormal enzyme functions, which, in turn, affect the energy metabolism of myocardial cells.

### 4.3. Limitations

Firstly, although the mice we used carry the same genetic mutations as humans, the conclusions of this study need to be further validated in human myocardial tissue due to ethnic differences. Secondly, a more in-depth analysis and further validation of key proteins as well as upstream and downstream regulatory mechanisms are required to identify the key signaling pathways and mechanisms leading to the occurrence of HOCM.

## 5. Conclusions

In this study, we used C57 mice with the same gene mutation as humans to detect lactylation that is more similar to the physiological and pathological states of humans. And we found that significant lactylation occurred in the proteins of the myocardial tissues of the HOCM and NOHCM mice, mainly including Acads, Acaa2, Mdh2, Myl2, and Myl3. Abnormalities of these proteins can lead to abnormalities in the energy metabolism of myocardial tissues in HOCM and may also be related to the impaired myocardial contractile function in HOCM. In this study, we selected NOHCM mice as the control group, which made up for the deficiency in previous studies, which mostly used WT mice as the control. Meanwhile, taking lactylation as the entry point, this study is one of the first studies on lactylation in HCM, laying a preliminary theoretical foundation for further research on the mechanism of HOCM.

## Figures and Tables

**Table 1:** Basic information and echocardiogram parameters of mice.

	WT Mice (*n* = 3)	HOCM Mice (*n* = 3)	NOHCM Mice (*n* = 3)	*p* Value
Body Weight (g)	23.47 ± 0.86	23.93 ± 1.65	24.23 ± 2.05	0.844
Heart Weight (mg)	69.70 ± 4.06	105.27 ± 2.80 ^^^	104.16 ± 0.90 *	<0.05
Tibia Length (mm)	25.13 ± 0.72	25.33 ± 1.00	24.60 ± 0.78	0.576
Heart Weight/Body Weight	2.97 ± 0.15	4.40 ± 0.23 ^^^	4.32 ± 0.37 *	<0.05
Heart Weight/Tibia Length	2.77 ± 0.09	4.24 ± 0.15 ^^^	4.16 ± 0.10 *	<0.05
IVSd ^a^ (mm)	0.61 ± 0.06	1.01 ± 0.05 ^^^	0.82 ± 0.04 *^#^	<0.05
MLVWT ^b^ (mm)	0.61 ± 0.06	1.05 ± 0.06 ^^^	1.11 ± 0.06 *	<0.05
Long Axis/Short Axis ^c^	1.18 ± 0.12	1.03 ± 0.05	0.96 ± 0.15	0.217
LVEF ^d^ (%)	51.45 ± 5.91	56.80 ± 5.21	70.33 ± 6.97 *^#^	<0.05
LVOT-PG ^e^ (mmHg)	1.50 ± 0.63	32.63 ± 16.69 ^^^	2.89 ± 1.31 ^#^	<0.05

WT vs. HOCM ^^^ *p* < 0.05; WT vs. NOHCM * *p* < 0.05; HOCM vs. NOHCM ^#^ *p* < 0.05. ^a^ IVSd, interventricular septum diameter in end-diastole; ^b^ MLVWT, maximum wall thickness of the left ventricle; ^c^ long axis/short axis, the long axis length of the left ventricle/short axis of the left ventricle; ^d^ LVEF, left ventricular ejection fraction; ^e^ LVOT-PG, left ventricular outflow tract pressure gradient.

## Data Availability

The data presented in this study are available on request from the corresponding author.

## Supplementary Materials

The following supporting information can be downloaded at: https://www.mdpi.com/article/10.3390/bioengineering12040379/s1, Figure S1 (A) Pearson’s correlation coefficient (PCC) analysis. (B) Pareto-scaled principal component analysis (PCA) analysis. Experimental procedure for protein extraction, trypsin digestion, affinity enrichment, liquid chromatography-tandem mass spectrometry analysis, database search, and co-inmunoprecipitation.
