# Supplementary material for: Altered Lactylation Myocardial Tissue May Contribute to a More Severe Energy-Deprived State of the Tissue and Left Ventricular Outflow Tract Obstruction in HOCM
Source: Bioengineering (Basel). 2025 Apr 3;12(4):379. doi: 10.3390/bioengineering12040379 (PMC12024552; doi:10.3390/bioengineering12040379)
Supplement: Supplementary file 1 [file bioengineering-12-00379-s001.zip › bioengineering-3476585-supplementary.pdf]

Supplementary Materials

Protein Extraction

The sample was grinded with liquid nitrogen into cell powder and then transferred to a 5mL centrifuge tube. After that, four volumes of lysis buffer (1% Triton X-100, 1% protease inhibitor cocktail) were added to the cell powder, followed by sonication three minutes on ice using a high intensity ultrasonic processor (Scientz). The remaining debris was removed by centrifugation at 12,000g at 4°C for 10min. Finally, the supernatant was collected and the protein concentration was determined with BCA kit according to the manufacturer's instructions.

Trypsin Digestion

The sample was slowly added to the final concentration of 20% (m/v) TCA to precipitate protein, then vortexed to mix and incubated for 2h at 4 °C. The precipitate was collected by centrifugation at 4500g for 5 min at 4°C. The precipitated protein was washed with pre-cooled acetone for 3 times and dried for 1min. The protein sample was then redissolved in 200mM TEAB and ultrasonically dispersed. Trypsin was added at 1:50 trypsin-to-protein mass ratio for the first digestion overnight. The sample was reduced with 5mM dithiothreitol for 30min at 56°C and alkylated with 11mM iodoacetamide for 15min at room temperature in darkness. Finally, the peptides were desalted by Strata X SPE column.

Affinity Enrichment

To enrich modified peptides, tryptic peptides dissolved in NETN buffer (100mM NaCl, 1mM EDTA, 50mM Tris-HCl, 0.5% NP-40, pH 8.0) were incubated with pre-washed antibody beads (Lot number xxx, PTM Bio) at 4°C overnight with gentle shaking. Then the beads were washed for four times with NETN buffer and twice with H_2O. The bound peptides were eluted from the beads with 0.1% trifluoroacetic acid. Finally, the eluted fractions were combined and vacuum-dried. For LC-MS/MS analysis, the resulting peptides were desalted with C18 ZipTips (Millipore) according to the manufacturers' instructions.

Liquid chromatography-tandem mass spectrometry analysis

The tryptic peptides were dissolved in solvent A, directly loaded onto a home-made reversed-phase analytical column (25-cm length, 100μm i.d.). The mobile phase consisted of solvent A (0.1% formic acid, 2% acetonitrile/in water) and solvent B (0.1% formic acid in acetonitrile). Peptides were separated with following gradient: 0-18 min, 6%-22%B; 18-22min, 22%-30%B; 22-26min, 30%-80%B; 26-30min, 80%B, and all at a constant flow rate of 500 nl/minon a NanoElute UHPLC system (Bruker Daltonics). The peptides were subjected to capillary source followed by the timsTOF Pro mass spectrometry. The electrospray voltage applied was 1.6 kV. Precursors and fragments were analyzed at the TOF detector. The timsTOF Pro was operated in data independent parallel accumulation serial fragmentation (dia-PASEF) mode. The full MS scan was set as100-1700（MS/MS scan range） and 8PASEF (MS/MS mode) - MS/MS scans were acquired per cycle. The MS/MS scan range was set as 425-1025 and isolation window was set as 25m/z.

Database Search

Building the Spectral Library: The DDA data were processed using Spectronaut (v.17.0) software coupped with Pulsar search engine. Tandem mass spectra were searched against Mus_musculus_10090_SP_20230103.fasta (17132 entries) concatenated with reverse decoy database. The max missing cleavages was set as 2. Carbamidomethyl on Cys was specified as fixed modification. Lactylation was specified as variable modifications. False discovery rate (FDR) of protein, peptide and PSM was adjusted to <1%. The corresponding spectral library was imported into Spectronaut (v.17.0) software to predicts the retention time by nonlinear correction and searched against with DIA data.

Co-inmunoprecipitation

A. Preparation of the Immune Complex Note:

The amount of sample needed and the incubation time are dependent upon each

specific antibody-antigen system and may require optimization for maximum yield. The following protocol is for 2-10μg of affinity-purified antibody and can be scaled up as needed.

1. Combine cell lysate with 2-10μg of IP antibody per sample in a microcentrifuge tube. The suggested amount of total protein per IP reaction is 500-1000μg, as determined by the Pierce BCA Protein Assay.

2. Dilute the antibody/lysate solution to 500μL with IP Lysis/Wash Buffer.

3. Incubate for 1-2 hours at RT or overnight at 4ºC to form the immune complex.

B. Manual Immunoprecipitation Note:

To ensure bead homogeneity, mix the vial thoroughly by repeated inversion, gentle vortexing or using a rotating platform.

1. Place 25μL (0.25mg) of Pierce Protein A/G Magnetic Beads into a 1.5mL microcentrifuge tube.

2. Add 175μL of IP Lysis/Wash Buffer to the beads and gently vortex to mix.

3. Place the tube into a magnetic stand to collect the beads against the side of the tube. Remove and discard the supernatant.

4. Add 1mL of IP Lysis/Wash Buffer to the tube. Invert the tube several times or gently vortex to mix for 1 minute. Collect beads with magnetic stand. Remove and discard the supernatant.

5. Add the antigen sample/antibody mixture (Section B) to the tube containing pre-washed magnetic beads and incubate at room temperature for 1 hour with mixing.

6. Collect the beads with a magnetic stand, remove the unbound sample and save for analysis.

7. Add 500μL of IP Lysis/Wash Buffer to the tube and gently mix. Collect the beads and discard the supernatant. Repeat this wash twice.

8. Add 500μL of ultra pure water to the tube and gently mix. Collect the beads on a magnetic stand and discard the supernatant.

9. Low-pH Elution: Add 100μL of Elution Buffer to the tube. Incubate the tube at RT with mixing for 10 minutes. Magnetically separate the beads and save the supernatant containing the target antigen. To neutralize the low pH, add 10μL of

Neutralization Buffer for each 100µL of eluate.

Alternative Elution: Add 100µL of Lane Marker Sample Buffer (diluted five-fold with purified water) to the tube and heat the samples at 96-100ºC in a heating block for 10 minutes. Magnetically separate the beads and save the supernatant-containing target antigen.

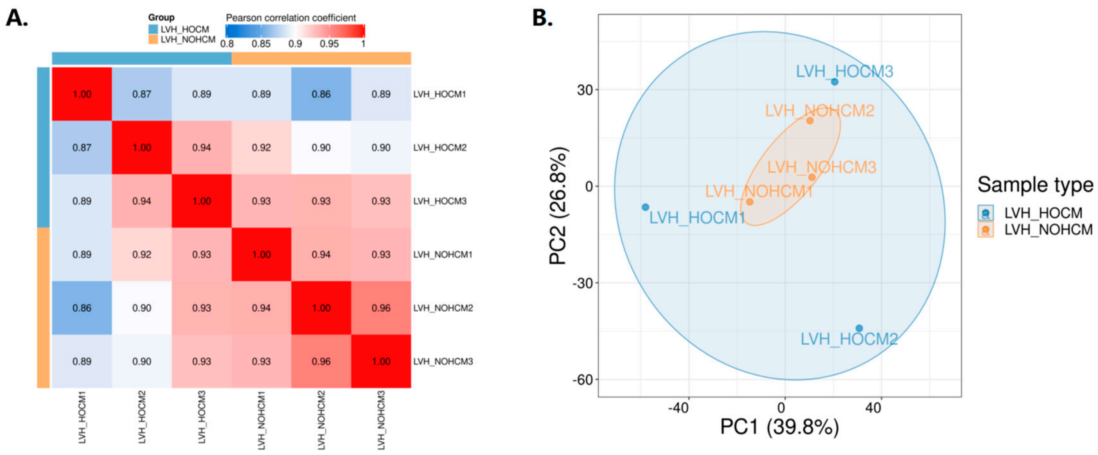

Figure S1 (A) Pearson's correlation coefficient (PCC) analysis. **(B)** Pareto-scaled principal component analysis (PCA) analysis.
